# Eosinophilia: Blood Films and the Differential Leucocyte Count

**Published:** 1907-04-06

**Authors:** 


### SPECIAL ARTICLE.

### EOSINOPHILIA: BLOOD FILMS AND THE DIFFERENTIAL LEUCOCYTE COUNT.

The term eosinophilia is used to denote any condition of the blood in which the coarsely granular eosinophile white corpuscles are relatively more numerous than they should be. To determine the fact *a differential leucocyte count* is required, and the method of doing this is as follows:—

In the first place, blood films must be made from a drop of blood usually obtained by pricking the lobule of the patient's ear with a sharp surgical needle. There are three different methods of spreading the films, namely:—

1. *The cigarette paper method.*—A piece of clean ungummed cigarette paper is cut the same width as the microscope slide; the slide itself is made absolutely clean; a small drop of blood is collected at one end of the slide by holding the latter up to the lobule of the ear where this has been pricked; the slide is laid upon the table, and the narrower edge of the cigarette paper is placed over the blood drop on the slide while the other end of the cigarette paper is held up so that the paper itself forms an angle of about 35° with the slide; the blood drop will now be lying in the angle between the cigarette paper above and the slide below, and by keeping this angle about 35° and drawing the cigarette paper steadily but fairly rapidly along the surface of the slide, a smooth and thin film of blood will be left upon the latter. The film should be just so thick that it dries within a minute of being spread; if thicker, drying will be so long delayed that the corpuscles will have run into rouleaux and also have become deformed. Never smear the same film twice. If the first film be too thick make a new one, using a rather smaller drop of blood to start with; it is also possible to vary the thickness of the film by varying the angle at which the cigarette paper meets the slide; by making the angle greater than 35° the film will be thinner, and *vice versâ*.

2. *The method of slipping two slides apart.*—In using this method two microscope slides should be made perfectly clean. A drop of blood is received upon the centre of one of them, not at the end as in the cigarette paper method; the second slide is then laid flat upon the first, over the blood drop, so that the latter spreads out in a thin layer between the two. The pair of slides is then held with the thumb upon the upper surface of the top one and the index finger on the under surface of the lower one, and by a movement of the thumb in one direction and the index finger in the other the two slides are made to glide over one another smoothly and evenly from end to end until they are completely separated; in this way a film is made on each slide at once.

3. *The method of pushing the blood drop along the surface of one slide by means of the edge of another slide.*—This is the simplest and best of the three methods, though it is easier to demonstrate than to describe. Two microscope slides are made perfectly clean; a drop of blood is received upon one, near one end; the edge of the other is then laid at right angles across the drop of blood in such a way that this second slide forms an angle of about 140° with the first and is evenly in contact with it at this angle. The upper slide is then steadily and smoothly pushed along the surface of the other from end to end, the blood drop being thereby pushed along it at the same time and leaving in its train a perfectly thin and even blood film. When a film has thus been made upon the first slide by means of the edge of the second, a similar film, from a new drop of blood, can be made upon the surface of the second slide by means of the edge of the first.

It will be noted that we recommend the making of films upon microscope *slides* in all cases, and not on cover slips. The reason is that, for purposes of examination with a $\frac{1}{12}$th inch oil immersion lens, there is no need to use canada balsam and cover glass at all; the cedar wood oil is put directly on to the film, and makes the preparation quite as transparent and twice as easy to examine as do canada balsam and a cover glass. Moreover, cover glasses are much more liable to be broken than are slides, and they are much more difficult to clean.

Before examining the films, however, it is necessary to fix and stain them. By far the most convenient and rapid method of doing this is to use Jenner's stain, which fixes the films at the same time as it stains them. The busy practitioner will usually prefer to send his films to a clinical laboratory both for staining and examination and report, but in case he should wish to stain and examine his own films we give the details of the procedure. The formula for Jenner's stain is as follows:—

| | |
|---|---|
| Crystalline methylene-blue and eosine... | 0.5 gram |
| Methyl alcohol ... ... ... ... | 100 c.c. |

It is sold by most large manufacturing chemists ready for use. Enough is required to nearly fill a cylindrical glass jar sufficiently deep to take the microscope slides vertically. As soon as the film is dry it may be put into the stain in the jar, though the staining may be postponed for almost any length of time without detriment to the films if they are protected from the dust. It usually suffices to leave them in the stain for five minutes, though a little longer will do no harm; and then comes the crucial point upon which the success of the staining, or t[illegible] reverse, depends—namely, the washing of the film[illegible] This must be done in distilled water; but ma[illegible] samples of even distilled water contain enou[illegible] ammonia to render the reaction alkaline, a[illegible] thereby damage the stain. The easiest way [illegible] obviate this difficulty is to keep a special jar for [illegible] washing water, and before using the latter to a[illegible] to it a few drops of the Jenner's stain, just eno[illegible] to saturate the water with it. The ingredients [illegible] Jenner's stain are soluble in various alcohols, bu[illegible] does not take much to saturate the distilled water with them even if the water be slightly alkaline. This jar of water should not be thrown away after the washing of the films in it; but, dirty though it may appear to be, it should be kept for future film-washing just as it is. The films, after five minutes or a little longer, are lifted out of the jar of Jenner's stain, the excess of the latter is allowed to run back into its jar, and the films are then transferred to the washing jar and kept moving gently backwards and forwards in the stained water for about a minute. They are then lifted out, held vertically with their lower ends upon a piece of blotting-paper, which will suck up most of the water which can be drained off from them. They are then laid, film upwards, upon a clean dry surface, and either allowed to dry spontaneously or else dried rather more rapidly by blowing a current of air across them. This can be done by blowing with the mouth, or perhaps better by a rubber ball and tube like a barber's bay-rhum squeegee, with a glass pipette for a nozzle. On no account should the films be blotted, or portions of the blotting-paper will be very apt to remain on the slide, and, worse still, patches of the film may be carried off on the paper. Jenner's stain only fixes the films very lightly.

As soon as dry the film is ready for examination. Put a drop of cedar wood oil upon it, place it under the oil immersion lens, and proceed with the microscopical examination. It is a very great convenience to have a mechanical stage, but even without one a very good idea of the differential leucocyte count is possible. The principle of making this count is to examine consecutive white corpuscles, taking care not to examine the same part of the film twice; each corpuscle as it is seen is noted down under its appropriate kind, and when 100 leucocytes have been seen the observer will know how many of each kind there are in the 100. To make the differential count more accurate, 200 or more leucocytes would be examined in this way; but for clinical purposes the evidence afforded by the first 100 leucocytes is nearly always the same as that afforded by any larger number; moreover, the calculations are so much simplified if 100 be the number taken. With a mechanical stage one can be sure that the leucocytes are strictly consecutive, and the count is therefore of more certain value than when the slide is moved about erratically by hand.

In normal blood there are four distinctive kinds of white corpuscles, which at various times have received various names. The names themselves matter little, for the cells are so obvious as a rule that they might almost be termed A, B, C, and D respectively; but the names commonly used, and the relative proportions of the four kinds in normal blood, are as follows:—

| | | |
|---|---|---|
| Small lymphocytes ... ... ... | about 30 | per cent. |
| Large lymphocytes (or hyaline) ... | ,, 8 | ,, ,, |
| Polymorphonuclear cells (sometimes termed finely granular eosinophile cells)... ... ... ... ... | ,, 60 | ,, ,, |
| Coarsely granular eosinophile* cells | ,, 2 | ,, ,, |
| | 100 | |

* Eosinophile and oxyphile are synonymous terms.

In the differential leucocyte count the variations from the above proportions have to be *considerable* before any clinical significance can be attached to them; small variations are of no moment, clinically; for example, the polymorphonuclear cells may be as low as 55 per cent. or as high as 65 per cent., and yet the person be perfectly well.

In so brief an article as this it is not possible to give in detail the characters of each of these varieties of cells, but we may give them very shortly. In a film stained by Jenner's stain the *small lymphocytes* will have a thin rim of protoplasm stained pale blue, surrounding a perfectly *round nucleus* stained dark blue; the size of the cell varies considerably, but it is usually only a little larger than a red corpuscle. The *large lymphocyte* is a much bigger cell, with a considerable amount of clear pale blue protoplasm surrounding a *kidney-shaped nucleus* which stains but little deeper blue than does the protoplasm; some small lymphocytes look nearly large enough to be large lymphocytes, but if the nucleus is round the chances are that the cell is a small lymphocyte which has become much flattened out in making the film rather than a large lymphocyte. The *polymorphonuclear cell* has a blue nucleus which is quite irregular, with swellings in it which may possibly suggest that more than one nucleus is present; on careful examination these swellings of the nucleus are seen to be connected together by bridles of nuclear tissue, and, roughly speaking, the outline of the many-lobed nucleus is horse-shoe shaped; the protoplasm stains pink, and in the protoplasm there are innumerable very small pink granules; it is from the latter that the cells are sometimes termed "finely granular eosinophile," and at first there is a tendency to regard these cells as *coarsely granular eosinophile cells*; the latter, however, when once seen, can never be confused with the polymorphonuclear cells because the pink granules in these coarsely granular eosinophile cells are very much larger, though in the shape of the nucleus and in other respects the two cells are alike. The large pink granules cannot be mistaken, and whenever, in examining a film, there is doubt as to whether a particular white corpuscle is a polymorphonuclear cell or a coarsely granular eosinophile cell, it is almost certainly the former.

The original purpose of this article was to discuss the conditions under which the coarsely granular eosinophile cells of the blood are increased in numbers relatively to the other kinds of leucocytes; that is to say, the conditions in which the presence of eosinophilia may be of value as a means of differential diagnosis. We will now do so.

In normal persons these eosinophile corpuscles seldom exceed 2 per cent. in the differential leucocyte count; but it occasionally happens that persons who seem to be in perfect health have as many as 5 per cent. of eosinophile corpuscles. When there are more than 5 per cent. there is definite eosinophilia. There are three main groups of conditions in which eosinophilia occurs, and these are:—(1) Spasmodic asthma; (2) certain skin diseases; (3) certain parasitic infections. We will discuss each in turn.

### 1. Spasmodic Asthma.

The different varieties of spasmodic or paroxysmal dyspnœa are numerous, and will form the subject of a future article. All of them may resemble asthma more or less, but the form which is most difficult to distinguish from true asthma is that which is sometimes called bronchitic asthma. Bronchitic asthma is not asthma at all, but bronchitis with more or less emphysema. The reason why the diagnosis between the two is so difficult is that spasmodic asthma causes emphysema, and this in turn leads in time to bronchitis, so that bronchitis and emphysema become superimposed upon the spasmodic asthma, and it may become extremely difficult to tell whether the trouble is primarily bronchitic, or primarily true asthma and secondarily bronchitic. In the spasmodic affection there is no inflammation to start with; in the bronchitic, as the name implies, inflammation of the bronchi is the essential lesion. The treatment of true asthma is obviously different to the treatment of bronchitis, so that it is important to know which predominates in a given case. The history may help the diagnosis; for example, the patient may say that the attacks date from youth, and in the earlier years of the trouble the attacks consisted of dyspnœa only, without cough. This would be in favour of true asthma with superimposed bronchitis, for in spasmodic asthma there is very little cough except at the end of an attack. The history, however, may not be clear; moreover, the attacks may be primarily asthmatic, and yet may not have begun till middle age. The physical signs will help but little, for in both cases the chest will show the so-called barrel-shape of emphysema, and all sorts of rhonchi will be heard in the lungs. An additional means of diagnosis is valuable, and we have such additional means in eosinophilia. In spasmodic asthma the coarsely granular eosinophile cells of the blood are commonly increased during the time of the paroxysms, the eosinophilia disappearing between the attacks. In bronchitis, on the other hand, there is seldom eosinophilia. A differential leucocyte count may therefore decide between the two. The eosinophilia in true asthma may be slight, or it may be very marked. In many cases the coarsely granular eosinophile cells are from 6 per cent. to 10 per cent. of all the leucocytes; in a few cases their proportion is very much greater, up to as much as 60 per cent. in the differential count.

As an instance of the value eosinophilia may have in deciding important issues may be cited the case of a gentleman who was in one of the public services abroad. He was married and had two children; his sole source of income was from the service, and in due course he was to get a pension. He developed acute attacks of dyspnœa, and the authorities decided that he either had a thoracic aneurism or else was suffering from asthma. If he had an aneurism he was to be invalided from the service, with poverty before him; if it be asthma, he was to be allowed to continue his work with the prospect of a pension. He was sent home to England for the diagnosis to be settled, and opinions were again divided between aneurism on the one hand and asthma on the other. Skiagrams were indefinite, and there seemed little hope of deciding which of the two things was the matter until a differential leucocyte count showed that 59 per cent. of his white corpuscles were coarsely granular eosinophile cells. This decided the difficulty in favour of asthma, and the gentleman is still in the service and well off, instead of invalided and in poverty.

### 2. Skin Eruptions.

It is often stated that many skin eruptions may give rise to eosinophilia. This is academically true, but in very few skin diseases is it the rule rather than the exception for eosinophilia to occur. In cases of eczema, psoriasis, impetigo, herpes zoster, ringworm, seborrhœic dermatitis, acne, lichen-planus, and so on, a patient here and there may show slight eosinophilia; in the great majority of cases, however, the coarsely granular eosinophile cells show no departure from the normal. There are only two skin diseases which are at all constantly associated with eosinophilia, and these are *pemphigus* and *herpes gestationis*. Even with these there are cases without eosinophilia; it is, however, the rule rather than the exception for pemphigus and herpes gestationis to be accompanied by eosinophilia. Neither condition is common, but every practitioner will see one or other from time to time; and in each there may be difficulty in diagnosing it from other vesicular or bullous eruptions, such as drug rashes, artefacts, and so on. The presence of eosinophilia will be a considerable argument in favour of pemphigus or herpes gestationis (Dühring's disease) as the case may be.

### 3. Parasitic Affections.

It is not every parasitic affection that is associated with eosinophilia. For example, it does not occur in—

(1) Parasitic skin affections.
- (*a*) Vegetable:
  - Tinea tonsurans.
  - Tinea circinata.
  - Favus.
  - Tinea versicolor.
- (*b*) Ancinal:
  - Pediculosis capitis.
  - Pediculosis corporis.
  - Pediculosis pubis.
  - Scabies.
  - Demodex folliculorum.

(2) The milder intestinal parasitic infections:
- Ascaris lumbricoides.
- Oxyuris vermicularis.
- Trichocephalus dispar.

(3) Malaria.

On the other hand, it does occur, sometimes in very marked degree, in cases of infection with the following parasites:—

| | |
|---|---|
| Tænia solium<br>Tænia mediocanellata<br>Bothriocephalus latus | Moderate eosinophilia, *e.g.* 6% to 10% |
| Bilharzia hæmatobia<br>Trichina spiralis<br>Anchylostomum duodenale<br>Filaria parasites (with or without elephantiasis) | Considerable eosinophilia as a rule, from 10% to 60% |

It is true that in many cases no eosinophilia is needed to assist the diagnosis; the latter may be obvious, from the passage of the tape-worm segments per anum, for example, or from the presence of the typical Bilharzia ova in the urine, and so on. Nevertheless, the eosinophilia may occasionally be of great assistance. For instance, in the Cornish tin-mines there were large numbers of cases of obscure anæmia, severe enough to prove fatal, the diagnosis of the cause of which was not made for years. A government inquiry found that these were almost all cases of ankylostomiasis, with eosinophilia; had the eosinophilia been examined for in the first instance its presence would have suggested the possibility of parasitic infection long before, and ankylostomiasis confirmed by examination of the stools for parasites and ova. Moreover, prophylactic measures against the spread of the infection might have been adopted sooner; and those persons who were already infected would have received thymol in five to ten grain doses, which is the cure for the disease.

One more example of the possible value of eosinophilia may be of interest, and that is in connection with Bilharzia hæmatobia. After the South African war numbers of men returned to all parts of Great Britain suffering from hæmaturia and consequent anæmia, albuminuria, and in a good many cases œdema of the legs. At first sight the diagnosis in some of these cases looked like subacute nephritis, and unless care were taken treatment for nephritis might easily be adopted—that is to say, prolonged rest in bed, strict milk diet, liquor ammoniæ acetatis, and pulv. jalapœ co. Examination of the urine for renal tube casts often led to the discovery of Bilharzia ova, so that the correct diagnosis was soon made. But it is well known that Bilharzia ova are not necessarily found in the first specimen of urine examined; in one case at least, and no doubt in others, no ova were found at the usual routine examination at the start, and as there were many epithelial cells as well as red blood discs in the urine the condition was thought to be nephritis. A blood film, however, showed 14 per cent. of coarsely granular eosinophile corpuscles; parasitic infection was suspected, further microscopic examinations of the urine were made, and at last the Bilharzia ova were found. In this patient the eosinophilia led to the treatment being quite changed; it was no longer necessary to keep him strictly in bed, he was able to be up and about seeing to his own affairs, and a generous meat diet took the place of the strict milk diet that had previously seemed essential.

